# Educational Approach: Application of SWOT Analysis for Assessing Entrepreneurial Goals in Senior Dental Students

**DOI:** 10.3390/ejihpe14030049

**Published:** 2024-03-20

**Authors:** Maria Antoniadou, Antonia Kanellopoulou

**Affiliations:** 1 Department of Dentistry, School of Health Sciences, National and Kapodistrian University of Athens, 11527 Athens, Greece; akanel@dent.uoa.gr; 2 Certified Systemic Analyst Professional, Executive Mastering Program in Systemic Management, University of Piraeus, 18451 Piraeus, Greece

**Keywords:** SWOT analysis, competitive healthcare sector, senior dental students, dentists, internal strengths, eaknesses, external opportunities and threats, educational tool, career decision-making, dental entrepreneurship, career outcomes, career counselors

## Abstract

The SWOT (strengths, weaknesses, opportunities, and threats) analysis is a framework used to evaluate a company’s competitive position and to develop strategic planning. In the competitive dental sector, it can aid dentists in identifying and analyzing internal strengths and weaknesses, as well as external opportunities and threats. This study focuses on senior dental students of the Department of Dentistry at the National and Kapodistrian University of Athens, Greece, aiming to scrutinize their use of SWOT analysis and assess its application as a tool for evaluating entrepreneurial goals and making career decisions in dental entrepreneurship. The research sample comprises 116 senior dental students (N1) in the final undergraduate year of their dental education, with data collection accomplished through the administration of an e-questionnaire during the obligatory course of “Organization and management of dental practices” in December 2023. The data extracted from the SWOT analysis encompass internal and external factors, gender distinctions, and outcomes derived from Stepwise Binary Logistic Regression concerning predictor markers. The results from the SWOT analysis of 114 valid questionnaires (N2), revealed that participants identified communication skills (50%) and organization skills (49.10%) as their primary strengths, followed by favorable personal traits contributing to goal success (36%). Weaknesses predominantly centered around emotional and personal traits like anxiety (41.20%) and other characteristics, alongside practical challenges such as lack of initial capital (24.60%). Main opportunities included collaboration with experienced dentists (33.30%) and access to training programs (27.20%), while economic instability in Greece (77.20%) and the saturated dentist profession (26.30%) were perceived as significant threats. Gender differences were notable, with female dentists more likely to report organization skills as a strength and anxiety as a weakness. Values such as industriousness, persistence, and ethics were commonly shared, with actions focusing on training programs (57.9%) and gaining experience with experienced dentists (29.8%). Cluster analysis identified two subgroups, with one emphasizing utilizing all available options (*n* = 49) and the other prioritizing on gaining professional skills and experience (*n* = 65). Logistic regression indicated that participants valuing industriousness were less likely to explore all available options, while those recognizing personal traits were more likely to do so. The study’s outcomes highlight key predictor factors linked to a proactive orientation in career decision-making among senior dental students. These insights offer valuable implications for educational institutions and career counselors.

## 1. Introduction

In the dynamic landscape of the healthcare industry, where market demands, technological advancements, regulatory changes, and patient expectations constantly evolve, strategic planning and decision-making are pivotal for success [1,2,3]. The SWOT analysis, (strengths, weaknesses, opportunities, and threats), a fundamental framework widely used in healthcare organizations, provides a structured methodology for assessing internal strengths and weaknesses, as well as external opportunities and threats [1,3,4]. This tool’s application is particularly valuable for crafting informed strategies in the healthcare sector, allowing organizations to adapt to the ever-changing environment [5,6]. Dentists and dental practices can also benefit significantly from the application of SWOT analysis, especially concerning various aspects of their business and professional practice [7].

In the field of dental education, SWOT analysis further serves several crucial purposes. Firstly, it aids in identifying personal strengths and weaknesses among dental students, helping them recognize their strengths while addressing areas needing improvement. This process aligns with the educational aspect of dental training, where students actively engage in self-assessment and skill development [8]. Secondly, it facilitates the recognition of opportunities for growth and development within dental education, such as research projects, community outreach programs, and specialized training, all of which contribute to a holistic educational experience [9]. Thirdly, SWOT analysis helps in acknowledging threats and challenges students may face, enabling them to prepare and strategize effectively. Furthermore, it assists in career planning and decision-making by aligning students’ strengths with suitable career paths and guiding them in addressing weaknesses through further education or mentorship. Lastly, SWOT analysis enhances personal development and improvement strategies by guiding students in designing action plans to enhance their skills and capitalize on their strengths [9].

According to these purposes, for senior dental students who are on the verge of making crucial career decisions, the utilization of SWOT analysis becomes imperative. As the landscape of dental healthcare undergoes transformation post-COVID-19, there seems to be a need for integrating innovative approaches and entrepreneurial skills into dental education to prepare students for adapting to changing circumstances and fostering creativity in dental practice [1]. However, the transition to entrepreneurship demands careful consideration of various factors to ensure long-term success. Senior dental students possess a unique set of strengths derived from their educational background, clinical skills, and hands-on experience gained during their studies [10]. Yet, potential weaknesses may stem from limited business acumen and financial management skills, or a lack of experience in practice management [11].

While SWOT analysis has been employed already in various capacities within dentistry [7,8,9,10,11], its novel application for assessing dental students’ anticipation of their future professional trajectories constitutes a noteworthy contribution. This research investigates the application of SWOT analysis among senior dental students at the Department of Dentistry, National and Kapodistrian University of Athens, Greece. Emphasis is then placed on evaluating their preparedness to initiate independent dental practices shortly after graduation, offering valuable insights into the distinct requirements of senior dental students during the decision-making process [1,9]. Simultaneously, the study aims to investigate the relevance of SWOT analysis in the realm of dental entrepreneurship. Its objective is to provide comprehensive insights and guidance on the effective utilization and seamless integration of SWOT analysis into decision-making processes during the transitional phases between the academic and professional life of dental students, addressing the relevant gap in the existing literature. The overarching goal is to empower senior dental students with strategic insights that can elevate their decision-making process and augment the likelihood of success in their entrepreneurial pursuits.

## 2. Materials and Methods

### 2.1. Sample of the Study

The study sample consisted of 116 senior dental students (N1 = 116), all currently enrolled in the Department of Dentistry at the National and Kapodistrian University of Athens, Greece, and actively engaged in the “Organization and management of dental practices” course for the academic year 2023–2024. The course is an integral component of the dental curriculum, occurring during the ninth semester of their undergraduate studies. These participants are in the final stages of their dental education, nearing completion of their academic requirements and preparing for graduation. All 116 senior students (43 male, 73 female) on the course were asked to participate.

### 2.2. Ethics Statement

This research adheres to the principles of ethical conduct in research involving human subjects and is conducted in accordance with the guidelines outlined in the Declaration of Helsinki. The study received ethical approval from the Institutional Review Board (IRB) of the department of Dentistry, National and Kapodistrian University of Athens, Greece, concerning inquiries conducted as part of the course, with one of the authors of the present study assuming responsibility for the course for the academic year 2023–2024 (618-20/12/2023). Participants were fully informed about the nature, purpose, and procedures of the research before their involvement and were also introduced to the SWOT tool during the consecutive seminar where thorough discussion on all parts of the tool was followed. They had also received a detailed written and verbal explanation of the study and the SWOT tool and were provided with an informed consent form outlining their voluntary participation, the potential risks and benefits of involvement, the confidentiality of their data, the right to withdraw from the study at any time without consequence and the absence of rewards. Completing and submitting the questionnaire served as confirmation of the participant’s willingness to take part in the study. Strict confidentiality measures were in place, ensuring the anonymity of participants and that personal information will not be referenced or published.

### 2.3. Methods

This research adopts a questionnaire-based approach, a method commonly employed in healthcare studies for data collection on relevant themes, as evidenced by previous works [12]. The questionnaire was uploaded to Google Forms, providing students with a QR code for convenient access during completion via their smartphones during the ninety minutes long relevant seminar of the course. Instructions for completion were addressed in the preface of the form (Appendix A). 

The questionnaire of the study is structured into basically two parts (Appendix B): Part A had a question on the gender of participants (Q1) and Part B explored participants’ perspectives on establishing their own dental practice (Q2–Q8). More specifically, Q2–Q5 were open-ended, to allow students to provide as much detail as they wished in their responses and addressed strengths, weaknesses, opportunities, and threats of the students on their future dental entrepreneurship as suggested using SWOT analysis. Questions Q6 and Q7 were given even more interest on personal values and actions that students could incorporate to proceed to business after graduation (within the next three years). This approach was based on studies exploring the association between values and personality traits, suggesting how character strengths can be viewed as manifestations or expressions of underlying values. More precisely, it is reported that certain values, for example, gratitude and self-transcendence, align with specific personality traits, potentially shedding light on how individuals’ value systems contribute to the formation and expression of their personalities and to what they consider as their weaknesses or strengths [13,14]. Finally, question Q8 reported on the ease of applicability of the tool.

All questions were also obligatory to submit the form. The study employed quantitative analysis to scrutinize the qualitative responses gathered from Part B, focusing on the professional outlook of senior dental students. Qualitative methodologies like the one used in this study are reported to enhance the depth and richness of research findings in the healthcare field [15], especially dentistry [16]. The estimated time for participants to complete the questionnaire was 16–25 min.

### 2.4. Statistical Analysis

The data collected from the SWOT analysis were analyzed with the statistical package IBM SPSS v. 28. Respondents’ short answers in the open-ended questions of SWOT components (strengths, weaknesses, opportunities, threats, values, and actions) were analyzed thematically and absolute and relative frequencies (n, %) were reported per theme and SWOT component. Since the respondents did not respond with complete sentences but answered briefly, we used the adjectives and nouns that they have mentioned to form the categories. Their statements were clearly stated, and there were not such deep meanings that caused disagreements among analysts who both agreed in the categories setting.

Then, chi-square tests of independence were performed with the Fisher exact test correction when needed, to address potential differences between genders. To provide an overview of new dentists’ profiles in terms of their actions to achieve their goal, Two-Step cluster analysis was implemented with the various reported actions as predictors, extracting two subgroups [17]. To detect the most influential factors that separate the dentists’ action profiles, binary logistic regression analysis with stepwise backward elimination [18] was performed, with the dependent variable being the action clusters and the independent variables being the themes extracted from the SWOT components.

## 3. Results

The data extracted from the SWOT analysis are presented in Table 1. The response rate was 98.27% (114 correct questionnaires were finally collected out of 116 senior students, 43 males and 71 females). All participants willingly submitted the questionnaire explaining the high response rate of the study. In our analysis, personal traits were considered as characteristics primarily inherent to an individual’s personality, while skills were regarded as attributes that can be acquired through learning [19]. So, in our findings, an individual is considered industrious if he or she demonstrates perseverance and determination in performing a task and is in the spectrum of work ethic. It is already reported that industrious people are more likely to believe that hard work is a virtue [20]. Also, for assessing “persistence” in our study, we used the definition of the term as a “voluntary continuation of a goal-directed action in spite of obstacles, difficulties, or discouragement” [21].

In terms of strengths, participants indicated mostly their communication skills (50% of participants) and organization skills (49.10%), followed by their favorable personal traits that would help them succeed in their goal (36%), such as persistence, patience, attention to detail, consistency, decisiveness, and critical thinking. Knowledge in their field (26.30%), industriousness (24.60%) and practical/clinical skills (17.50%) were also reported as strengths.

Interestingly, when asked about weaknesses, participants focused on emotional and personal traits such as anxiety (41.20%) and other personal characteristics (30.70%), (i.e., short-temper, lack of patience, indecisiveness), followed by lack of initial capital (24.60%), organization difficulties (16.70%), lack of experience compared to other dentists (14.00%) and perfectionism (10.50%). The main opportunities were considered as the possibility of collaboration with experienced dentists in dental practice (33.30%), access to training and specialization programs (27.20%), support from dentists or other physicians in the family (11.40%) and the opportunity to work as an intern in other dental offices to gain experience before opening their own practice (10.50%). The most important threats were described as the economic instability in Greece for the last 15 years (77.20%), the saturated profession of dentists in the country (26.30%), the high initial capital required for the equipment of the dental practice (23.70%), taxation rates in Greece (14.00%) and political instability in the Mediterranean region (13.20%).

Gender differences for the SWOT analysis components are presented in Table 2. The strength of organization skills was more likely reported by female dentists compared to male dentists (59.2% vs. 32.6%, respectively), while knowledge in their field was reported more frequently by male dentists (39.5% vs. 18.3%). Also, females noted their anxiety as a strong detriment to achieving their goal (49.3% vs. 27.9% for female and male participants, respectively). Female participants were more likely to consider access to training and specialization programs as an opportunity compared to males (35.2% vs. 14%), while support from dentists and other physicians in the family was considered an opportunity by 20.9% of male dentists and only 5.6% of female dentists. Economic instability was reported as a threat by female dentists (85.9%) and by male dentists (62.8%), while high taxation rates in Greece was considered a threat by 25.6% of male participants and only 7% of females.

Values and actions provided by the participants are summarized in Table 3, along with gender differences. Industriousness (24.6%), Persistence (35.1%) and Ethics (28.9%) were the main values reported by both male and female dentists. In terms of the path to achieve their goals, participation in training programs (57.9%), gaining experience by working with more experienced dentists (29.8%) and participation in scientific conferences (16.7%) were the most prevalent actions.

Actions were utilized in two-step cluster analysis and provided two subgroups (silhouette score of 0.2), namely the participants that intended to take advantage of all available options to achieve their goal (*n* = 49) and the ones that focused on gaining professional skills and experience (*n* = 65). Results of cluster analysis are presented in Table 4, showing that the first cluster included participants that intend to participate in training programs and scientific conferences, work as interns, pursue postgraduate degrees and utilize marketing strategies to promote their practice, while the second cluster included only participants that intend to gain practical experience and training.

In Table 5, the results of the Stepwise Binary Logistic Regression for the predictors of classification in cluster 1 “Taking advantage of all available options” are presented. Participants that report the strength of industriousness were less likely to take advantage of all the options to achieve their goals and were more likely to focus on gaining practical skills and experience (OR 0.338 95% CI 0.126–0.906). Moreover, participants that perceived their strength related to personal traits such as persistence, patience, attention to detail, consistency, decisiveness, and critical thinking were more likely to take advantage of all the options to achieve their goals (OR 2.922 95% CI 1.250–6.828).

The predictors included in stepwise backward conditional method: Strengths: Industriousness, Organization skills, Knowledge, Communication skills, Practical skills, Personal traits. Weaknesses: Anxiety, Personal traits, Lack of initial capital, Organization difficulties, Lack of experience or clinical skills, Perfectionism. Opportunities: Collaboration with experienced dentists in dental practice, Training/Specialization, Dentists/Physicians in the family, Work as an intern. Threats: Economic instability, Saturated profession, High initial capital, Taxation, Political instability.

**Figure 1:**
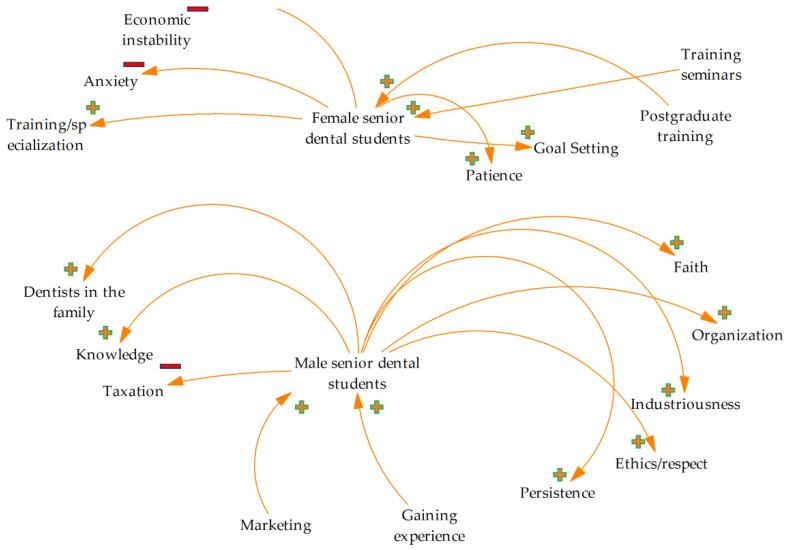
Schematic representation of factors influencing male and female students’ decision on dental entrepreneurship (factors are mentioned according to the highest prevalence) (−: factor affects negatively, +: factor affects positively).

A schematic representation of the data is seen in Figure 1.

Finally, the use of SWOT analysis in this educational approach for senior dental students was considered as helpful or very helpful by most of the sample (62.6%).

## 4. Discussion

Personal development plans play a pivotal role in enhancing self-development and professional advancement [21,22,23,24,25]. In this study, we report on data derived from the use of SWOT analysis in assessing goal setting for dental entrepreneurship in senior dental students of a dental department in a public university.

The Strengths analysis in our study had important feedback. Notably, industriousness emerged as a significant predictor, indicating that students with a strong work ethic are more likely to exhibit a proactive approach in utilizing available entrepreneurship opportunities in the market of dental services [26]. Moreover, personal traits, specifically strengths, were also identified as a significant predictor of career success in our study. This underscores the importance of self-awareness and using one’s unique qualities in career decision-making as also mentioned elsewhere [1,26]. The positive association between personal traits and the inclination to explore various options in dental entrepreneurship aligns with the idea that understanding one’s strengths contributes to effective decision-making and adaptability [27] and corresponds to a growth mindset [28]. Furthermore, the relevant literature underscores the influential role of mindset, specifically the distinction between fixed and growth mindsets, in shaping decision-making and career trajectories [29,30]. A fixed mindset, characterized by the belief in inherent limitations, leads individuals to perceive their abilities as rigid and resistant to change, impacting their reactions to unexpected career developments [19]. The skepticism and reluctance to adapt, which characterize the fixed mindset, become a self-fulfilling prophecy that discourages proactive engagement with career challenges as seen in our data [31]. In contrast, a growth mindset views abilities as malleable, embracing setbacks as opportunities for learning and improvement [26] as seen by most of our participants. Dental students with a growth mindset could approach unexpected developments such as challenges, fostering resilience and adaptability while in the university, as well as later in their profession [32,33]. This mindset encourages a proactive decision-making approach in our participants, marked by seeking feedback, experimenting with strategies, and ongoing development initiatives [34]. As discussed in Dweck’s concept of a growth mindset, fostering a growth mindset could be also vital for dental students, enabling them to navigate uncertainties and challenges with flexibility, open-mindedness, and a commitment to continuous learning [28]. The next step involves adopting an evolutionary mindset, using self-reflection, personal evolution, and addressing all opportunities for success [35] as seen for 43% of our participants too. An evolutionary mindset prompts individuals to explore competencies, talents, and ask challenging questions about their behavior, crucial for addressing complexities in dentistry [26,36]. In current and future high-performance dental environments, an evolutionary mindset will enhance adaptability, flexibility, and readiness for self-scrutiny and necessary changes, marking the path towards true leadership in the dental profession [37].

Although economic instability in the decision-making process of senior dental students in our study did not achieve conventional significance levels, its presence in the model suggests potential influence. This aligns with the broader recognition that economic considerations wield substantial influence on career decisions [38]. Also, our study confirms previous findings on gender-based differences in career decision-making, with female and male dental students showing distinct preferences in SWOT analysis components [39]. Female dentists emphasize organizational skills, while males prioritize technical proficiency and business acumen. We also identify new concerns, like taxation rates for male dentists, not previously discussed [39]. Additionally, marketing involvement positively influences our sample, consistent with other studies [40,41]. These findings report on the need for tailored educational approaches addressing gender-specific preferences and challenges in dental education and career expectations.

The Opportunities analysis was also important in our approach. The identification of key predictors opens avenues for targeted interventions and support mechanisms in dental education. Recognizing the significance of personal traits, educators and career counselors can design programs to enhance self-awareness and provide resources for students to better understand their strengths. Strengthening these personal attributes may positively influence career decision-making processes [42,43,44]. The study’s focus on opportunities aligns also with the broader context of career development theories, emphasizing the importance of identifying and capitalizing on favorable conditions [45]. The opportunities identified in the study, such as collaboration with experienced dentists and specialized training, suggest avenues for strategic career planning as discussed elsewhere [46]. Finally, threats were also searched in our study, with the overall political situation in the area being a significant factor for decision making for students, as also addressed elsewhere [47].

Senior dental students, as revealed by the literature and our study, prioritize attributes beyond goal setting in their hierarchy of decision-making factors [48]. Notably, characteristics such as persistence, ethics, respect, industriousness, organization, patience, and loyalty take precedence over goal setting in their considerations. This inclination suggests that senior dental students place substantial value on personal qualities and ethical principles when making professional decisions. While goal setting is acknowledged as a valuable aspect, its prioritization appears to be lower compared to these character-based attributes. Therefore, enabling senior dental students in effective professional decision-making involves fostering and emphasizing the cultivation of these ethical and character-driven qualities. This aligns with the notion that the decision-making process goes beyond merely setting goals and underscores the significance of a holistic approach that incorporates personal values and ethical considerations [49,50,51]. The identified factors encompass not only the personal attributes but also the importance of enhancing a classroom environment that encourages mastery goals and offers students opportunities to set their own goals. Additionally, accompanying goal setting with related steps such as planning, self-evaluation, feedback, and reflection emerges as crucial for a comprehensive decision-making process [52,53,54,55]. As Schunk [56] suggested “By themselves, goals do not automatically enhance learning and motivation.”

While the study offers valuable insights, it is essential to acknowledge certain limitations too. The non-significant association of certain variables, like economic instability and gender, underscores the complexity of career decision-making, potentially influenced by factors beyond those examined in this study [47,48]. Also, conducted solely in one institution, this study may not fully capture the perspectives, threats, and opportunities of all dental students. Additionally, while SWOT analysis offers simplicity, it might oversimplify career decision-making processes [2,21,46]. Subjective judgments and desirability bias could further affect the accuracy of self-reported data, moving responses towards societal expectations [57]. We should also consider the possibility that participants may present their choices favorably, masking uncertainties [58]. Despite providing a snapshot, the static nature of SWOT and a limited questionnaire time constrain a comprehensive understanding of evolving career trajectories. Future research should employ longitudinal designs and broader participant samples to address these limitations, enhancing the validity of findings [59]. Triangulating data sources could further improve validity in similar studies [60].

In summary, the SWOT framework proves a valuable tool in guiding senior dental students through strategic planning, aligning strengths with opportunities, and proactively addressing weaknesses and threats [61,62]. As Mary Renault suggests, it is crucial to prepare for anticipated challenges to avoid unforeseen shocks [63]. Thus, integrating educational approaches that foster a growth and evolutionary mindset among dental students is imperative. This includes creating a learning environment that promotes self-awareness, values mistakes as opportunities for growth, and instills personal responsibility [64,65]. Strategies like feedback-rich assessments, reflective exercises, and mentorship programs can nurture this mindset, preparing future dentists for success in dynamic healthcare environments [63,66].

## 5. Conclusions

This study examines the role of SWOT analysis for dentists in the competitive healthcare sector, focusing on senior dental students’ career decisions in dental entrepreneurship. Participants identified communication and organization as strengths, while weaknesses included anxiety and lack of capital. Opportunities lay in collaboration and training, while threats were economic instability and industry saturation. Gender differences were noted, with industriousness valued, but some students were less exploratory. The study suggests promoting self-awareness and exploring options through targeted programs, contributing to career development discourse.

## Figures and Tables

**Table 1:** SWOT chart of the data provided by the sample (*N* = 114).

SWOT Results	
Strengths Communication skills (50.00%) Organization skills (49.10%) Personal traits ^1^ (36.00%) Knowledge (26.30%) Industriousness (24.60%) Practical skills (17.50%)	Weaknesses Anxiety (41.20%) Personal traits ^2^ (30.70%) Lack of capital (24.60%) Organization difficulties (16.70%) Lack of experience (14.00%) Perfectionism (10.50%) *Other:* Oversensitivity (7.90%), Fear of failure (7.00%), Lack of confidence (7.00%), Lack of extended social circle (5.30%), Financial management difficulties (5.30%)
Opportunities Collaboration with experienced dentists in dental practice (33.30%) Training/Specialization (27.20%) Dentists/Physicians in the family (11.40%) Work as an intern (10.50%) *Other:* Financial support from family (7.90%), Extended social circle (7.00%), Increased demand for dentists (6.10%), Digital dentistry (4.40%), Retirement of dentists (3.50%), Dental tourism (0.90%)	Threats Economic instability (77.20%) Saturated profession (26.30%) High initial capital required (23.70%) Taxation (14.00%) Political instability (13.20%) *Other:* COVID-19 pandemic (5.30%), Bureaucracy (4.40%)

^1^: persistence, patience, attention to detail, consistency, decisiveness, critical thinking; ^2^: short-tempered, impatient, over-patient, indecisive.

**Table 2:** Gender differences for the SWOT analysis components.

	Total (*N* = 114)		Gender			
			Male (*n* = 43)		Female (*n* = 71)	
	n	%	N	%	N	%
*Strengths*						
Industriousness	28	24.6%	10 _a_	23.3%	18 _a_	25.4%
Organization skills	56	49.1%	14 _a_	32.6%	42 _b_	59.2%
Knowledge	30	26.3%	17 _a_	39.5%	13 _b_	18.3%
Communication skills	57	50.0%	22 _a_	51.2%	35 _a_	49.3%
Practical skills	20	17.5%	10 _a_	23.3%	10 _a_	14.1%
Personal traits ^1^	38	33.3%	12 _a_	27.9%	26 _a_	36.6%
*Weaknesses*						
Anxiety	47	41.2%	12 _a_	27.9%	35 _b_	49.3%
Personal traits ^2^	35	30.7%	14 _a_	32.6%	21 _a_	29.6%
Lack of initial capital	28	24.6%	10 _a_	23.3%	18 _a_	25.4%
Organization difficulties	19	16.7%	9 _a_	20.9%	10 _a_	14.1%
Lack of experience or clinical skills	16	14.0%	9 _a_	20.9%	7 _a_	9.9%
Perfectionism	12	10.5%	7 _a_	16.3%	5 _a_	7.0%
*Opportunities*						
Collaboration with experienced dentists in dental practice	38	33.3%	18 _a_	41.9%	20 _a_	28.2%
Training/Specialization	31	27.2%	6 _a_	14.0%	25 _b_	35.2%
Dentists/Physicians in the family	13	11.4%	9 _a_	20.9%	4 _b_	5.6%
Work as an intern	12	10.5%	3 _a_	7.0%	9 _a_	12.7%
*Threats*						
Economic instability	88	77.2%	27 _a_	62.8%	61 _b_	85.9%
Saturated profession	30	26.3%	15 _a_	34.9%	15 _a_	21.1%
High initial capital	27	23.7%	8 _a_	18.6%	19 _a_	26.8%
Taxation	16	14.0%	11 _a_	25.6%	5 _b_	7.0%
Political instability	15	13.2%	3 _a_	7.0%	12 _a_	16.9%

^1^ persistence, patience, attention to detail, consistency, decisiveness, critical thinking; ^2^ short-tempered, impatient, over-patient, indecisive. Note: Values in the same row not sharing the same subscript are significantly different at *p* < 0.05. Cells with no subscript are not included in the test. _a_, _b_: significant differences.

**Table 3:** Gender differences in values and actions reported by the sample.

	Total		Sex			
			Male		Female	
	n	%	N	%	n	%
*Values*						
Industriousness	28	24.6%	11 _a_	25.6%	17 _a_	23.9%
Persistence	40	35.1%	17 _a_	39.5%	23 _a_	32.4%
Patience	25	21.9%	9 _a_	20.9%	16 _a_	22.5%
Ethics/Respect	33	28.9%	13 _a_	30.2%	20 _a_	28.2%
Faith	15	13.2%	6 _a_	14.0%	9 _a_	12.7%
Organization	25	21.9%	10 _a_	23.3%	15 _a_	21.1%
Goal setting	13	11.4%	4 _a_	9.3%	9 _a_	12.7%
*Actions*						
Training/Seminars	66	57.9%	20 _a_	46.5%	46 _a_	64.8%
Gaining experience	34	29.8%	14 _a_	32.6%	20 _a_	28.2%
Participate in Scientific Conferences	19	16.7%	3 _a_	7.0%	16 _b_	22.5%
Postgraduate studies	17	14.9%	5 _a_	11.6%	12 _a_	16.9%
Marketing	13	11.4%	6 _a_	14.0%	7 _a_	9.9%

Note: Values in the same row not sharing the same subscript are significantly different at *p* < 0.05. Cells with no subscript are not included in the test.

**Table 4:** Two Step Cluster Analysis results for action profiles.

Predictors	Action Clusters			
	1 Taking Advantage of All Available Options (*n* = 49)		2 Focus on Professional Experience (*n* = 65)	
	N	%	n	%
Training/Seminars	27	55.1%	39	60.0%
Gaining experience	10	20.4%	24	36.9%
Participate in Scientific Conferences	19	38.8%	0	0.0%
Postgraduate studies	17	34.7%	0	0.0%
Marketing	13	26.5%	0	0.0%

**Table 5:** Results of Stepwise Binary Logistic Regression for the predictors of classification in cluster 1 “Taking advantage of all available options” (*n* = 49).

	B	S.E.	Wald	Df	p	OR	95% C.I.	
							Lower	Upper
Industriousness	−1.085	0.503	4.652	1	0.031	0.338	0.126	0.906
Personal traits (strengths)	1.072	0.433	6.130	1	0.013	2.922	1.250	6.828
Economic instability	0.767	0.515	2.216	1	0.137	2.152	0.784	5.905
Gender (Female vs. Male)	0.008	0.430	0.000	1	0.986	1.008	0.433	2.342
Constant	−1.012	0.752	1.812	1	0.178	0.364		

## Data Availability

The data presented in this study are available on request from the corresponding author.

## Appendix A

### Guidelines for Completion

The primary goal of this study is to contribute valuable insights to the dental education and entrepreneurship of senior dental students. In this research, all collected data will be treated with the utmost confidentiality, adhering to ethical guidelines and data protection regulations. Identifiable information will be stored separately, and access will be restricted to the research team to safeguard privacy. Any details (if any) that could potentially reveal participants’ identities will be anonymized or pseudonymized. Participation is entirely voluntary, and senior dental students have the right to withdraw without penalty, ensuring that their academic standing and relationship with the university remain unaffected.

Every effort will be made to minimize potential discomfort or inconvenience to participants, and the study poses no risks. All research data will be securely stored digitally, with restricted access limited to the authors. Data retention will follow ethical guidelines, and secure disposal will occur after a specified period.

For any questions or concerns, participants are encouraged to contact the principal investigator, or the Department of Dentistry of the National and Kapodistrian University of Athens’ Ethics Committee.

By submitting the questionnaire of the study, senior dental students consent to their participation and contribution in advancing knowledge on dental entrepreneurship. The research team is committed to upholding the highest ethical standards throughout the process.

The questionnaire consists of two parts: demographics (Part A) and questions exploring final-year dental students’ perspectives on their professional careers (Part B). It ensures anonymity, and no personal data is collected. Participation is voluntary, and there are no rewards. Students can complete the questionnaire once, implicitly accepting the rules of personal data protection. Full confidentiality is guaranteed, and personal information will not be mentioned in any reference or publication. Results will be used for scientific publications. Submission implies consent, and the estimated time for completion is a maximum of 25 min. We appreciate your cooperation. Thank you.

## Appendix B

The questionnaire of the study.

Q1. What is your sex?

Q2. SWOT analysis serves as a functional tool for scrutinizing goals, problems, or situations and formulating action plans or making informed decisions. Imagine you are strategizing for your upcoming dental practice, either upon completing your studies or within the next 2–3 years. To facilitate decisions aimed at realizing this goal, begin by enumerating the strengths of the project pertinent to you personally. Please highlight the skills and talents you currently possess or anticipate acquiring in the next three years. This could include clinical expertise, communication skills, leadership abilities, specialized training, or any other strengths that contribute to your success in dental practice.

Q3. Moving forward, please record the personal weaknesses you identify that may impede your ability to carry out the project. This encompasses defects, weaknesses, negative beliefs, psychological barriers, and familial or societal beliefs that may act as barriers to your professional goals. Consider areas where you may need further development or improvement, such as time management skills, clinical expertise in certain areas, communication challenges, or any other personal limitations that could hinder your progress.

Q4. Now, please document the threats present in the environment that you consider most important in posing a risk to your next professional steps. It is imperative to thoroughly consider these threats before making any decisions. This could include factors such as economic instability, competitive pressures, regulatory changes, technological advancements, or any other external challenges that may impact your ability to establish and maintain a successful dental practice.

Q5. Finally, please write down the opportunities present in the environment that could help you design your next professional steps. Consider potential avenues for growth and development, such as emerging market trends, advancements in dental technology, networking opportunities, community outreach programs, or any other favorable circumstances that could enhance your professional prospects. Identifying and capitalizing on these opportunities will enable you to make informed decisions and maximize your chances of success in your dental career.

Q6. Which specific personal values do you think will assist you in organizing your next professional goal?

Q7. Which actions do you believe will contribute to the attainment of your goal?

Q8. How beneficial was the use of the tool in designing your future professional steps?
